# Genome Sequences of Bacteriophages cd2, cd3, and cd4, which Specifically Target Carnobacterium divergens

**DOI:** 10.1128/MRA.00636-21

**Published:** 2021-08-26

**Authors:** Peipei Zhang, Angelle P. Britton, Kaitlyn A. Visser, Catherine A. Welke, Heather Wassink, Erica Prins, Xianqin Yang, Leah A. Martin-Visscher

**Affiliations:** a Agriculture and Agri-Food Canada, Lacombe, Alberta, Canada; b Department of Chemistry, The King’s University, Edmonton, Alberta, Canada; Portland State University

## Abstract

Carnobacteria have been implicated in food spoilage, but also in protection against pathogenic bacteria. We report the isolation and complete genome sequences of three bacteriophages (phages cd2, cd3, and cd4) that specifically target Carnobacterium divergens. The genome sizes are approximately 57 kbp and have limited homology to known enterococcal and streptococcal phages.

## ANNOUNCEMENT

Carnobacterium maltaromaticum and Carnobacterium divergens are lactic acid bacteria (LAB) commonly found in foods, particularly dairy, meat, fish, and shrimp (1, 2). Previously, it was thought that high bacterial loads of these organisms resulted in food spoilage (2, 3); however, recent reports suggest that the volatile organic compounds produced by carnobacteria have a negligible impact on food quality (4). Moreover, since carnobacteria produce antimicrobial peptides (bacteriocins) and organic acids, they can act as protective cultures by inhibiting the growth of food spoilage or pathogenic bacteria such as Listeria monocytogenes (5, 6). Within the food industry, bacteriophages pose a major threat to LAB that function as starter cultures for fermentation processes or protective cultures; as such, phages targeting LAB have been extensively studied (7–9). However, phages infecting carnobacteria are underrepresented in this field of study, and very few bacteriophages targeting *Carnobacterium* spp. have been reported (10, 11). Here, we report the complete genome sequences of three lytic bacteriophages (cd2, cd3, and cd4) that target various *C. divergens* strains.

Bacteriophages cd2 and cd3 were isolated from minced beef, and cd4 was isolated from ham, all purchased at a grocery store in Edmonton, Alberta, Canada (Table 1). In each case, a 1-g sample of meat was added to 10 ml of brain heart infusion broth (Bacto) and incubated overnight at 25°C. Following centrifugation (9,000 × *g*, 5 min, 4°C), the supernatant was filter-sterilized (0.2-μm filter) and used to prepare crude phage suspensions (11), using *C. divergens* LV13 (12) as the host strain. Purified suspensions of each bacteriophage were prepared using three consecutive rounds of single-plaque isolation using *C. divergens* LV13 as the host strain. Additionally, *C. divergens* B1 (6) was used to propagate and isolate phages cd2 and cd3.

**TABLE 1:** Characteristics of the assembled phage genomes

Isolate	Recovery source	Total no. of forward/reverse reads	Estimated coverage by trimmed reads (×)	Assembled genome size (bp)	GC content (%)	No. of contigs	No. of ORFs^*a*^	GenBank accession no.	
								SRA	Genome
cd2	Minced beef	94,533	594.7	57,220	39.0	1	111	SRR14848713	MZ398135
cd3	Minced beef	129,946	788.9	57,171	38.9	1	110	SRR14848712	MZ398136
cd4	Ham	97,629	604.2	56,713	38.7	1	109	SRR14848711	MZ399596

a ORFs, open reading frames.

Phage DNA was isolated using a proteinase K and SDS treatment, followed by phenol-chloroform extraction and ethanol precipitation (13). Libraries were constructed using a Nextera XT DNA library prep kit and sequenced using an Illumina MiSeq PE250 platform. Sequencing reads were trimmed using Trimmomatic v0.39 (14), where reads with an average quality score of a 4-base sliding window of <30 and length of <100 were subsequently removed. Genomes were assembled using SPAdes v3.14.0 (15) with kmers set at 21, 33, 55, 77, 99, and 127 bp. Contigs with a length of <500 bp or coverage of <10 were removed using a Python script (16). The genomes were predicted to be circularly permuted using PhageTerm v1.0.11 (17). Genomes were annotated with PHANOTATE v1.5.0 (18) using the default settings.

Table 1 lists the characteristics of the phage genomes. Using OAT v0.9 (19), the phages were found to have average orthologous nucleotide identities of 98.3 to 99.8% with each other. A BLASTN (20) search of the genomes against the nucleotide database in NCBI did not find closely related bacteriophages, as the top 10 matched nucleotide sequences displayed limited coverage (<5%) and identity (<75%). Analysis with ViPTree (21) suggests that the three phage strains belong to the *Siphoviridae* family and have limited homology to several enterococcal bacteriophages, including VD13 (22), vB_EfaS_IME198, IME-EF1 (23), SAP6 (24), BC-611 (25), and Streptococcus phage SP-QS1.

### Data availability.

Sequencing data for bacteriophages cd2, cd3, and cd4 are available in GenBank under BioProject number PRJNA738531. The accession numbers for the sequencing reads and genomes are listed in Table 1.
